# Genotoxicity and Immunotoxicity of Titanium Dioxide-Embedded Mesoporous Silica Nanoparticles (TiO_2_@MSN) in Primary Peripheral Human Blood Mononuclear Cells (PBMC)

**DOI:** 10.3390/nano11020270

**Published:** 2021-01-21

**Authors:** Luca Di Giampaolo, Gloria Zaccariello, Alvise Benedetti, Giulia Vecchiotti, Francesca Caposano, Enrico Sabbioni, Flavia Groppi, Simone Manenti, Qiao Niu, Anna Maria Giuseppina Poma, Mario Di Gioacchino, Claudia Petrarca

**Affiliations:** 1 Specialization School of Occupational Medicine, University G. d’Annunzio of Chieti-Pescara, I-66100 Chieti, Italy; luca.digiampaolo@unich.it; 2 Department of Molecular Sciences and Nanosystems and Centro di Microscopia Elettronica “Giovanni Stevanato”, Ca’ Foscari University of Venice, Via Torino 155/b, I-30170 Venezia-Mestre, Italy; gloria.zaccariello@unive.it (G.Z.); benedett@unive.it (A.B.); 3 Center of Advanced Sciences and Technologies (C.A.S.T.), University G. d’Annunzio of Chieti-Pescara, I-66100 Chieti, Italy; giulia.vecchiotti@guest.univaq.it (G.V.); francescacaposano@gmail.com (F.C.); enrico.sabbioni@mi.infn.it (E.S.); 4 Department of Physics, Università Degli Studi di Milano, Via Celoria 16, I-20133 Milano, Italy; flavia.groppi@unimi.it (F.G.); simone.manenti@unimi.it (S.M.); 5 Laboratorio Acceleratori e Superconduttività Applicata (LASA), Department of Physics, Università Degli Studi di Milano and INFN-Milano, Via F.lli Cervi 201, I-20090 Segrate, Italy; 6 Occupational Health Department, Public Health School, Shanxi Medical University, Taiyuan 030000, China; niuqiao55@163.com; 7 Department of Life, Health and Environmental Sciences, University of L’Aquila, I-67100 L’Aquila, Italy; annamariagiuseppina.poma@univaq.it (A.M.G.P.); digioacc@unich.it (M.D.G.); 8 Department of Medicine and Science of Ageing (DMSI), University G. d’Annunzio of Chieti-Pescara, I-66100 Chieti, Italy; 9 Institute of Clinical Immunotherapy and Advanced Biological Treatments, Piazza Pierangeli 1, 65121 Pescara, Italy; 10 Rectorate of Leonardo da Vinci Telematic University, Largo San Rocco 11, 66010 Torrevecchia Teatina CH, Italy

**Keywords:** nanoparticles, titanium oxide, Mesoporous Silica Nanoparticles, immunotoxicity, cytokines, cosmetic industry, UV filter, sunscreen

## Abstract

**Background:** TiO_2_ nanoparticles (TiO_2_ NPs) are the nanomaterial most produced as an ultraviolet (UV) filter. However, TiO_2_ is a semiconductor and, in nanoparticle size, is a strong photocatalyst, raising concerns about photomutagenesis. Mesoporous silica nanoparticles (MSN) were synthetized incorporating TiO_2_ NPs (TiO_2_@MSN) to develop a cosmetic UV filter. The aim of this study was to assess the toxicity of TiO_2_@MSN, compared with bare MSN and commercial TiO_2_ NPs, based on several biomarkers. **Materials and Methods:** Human peripheral blood mononuclear cells (PBMC) were exposed to TiO_2_@MSN, bare MSN (network) or commercial TiO_2_ NPs for comparison. Exposed PBMC were characterized for cell viability/apoptosis, reactive oxygen species (ROS), nuclear morphology, and cytokines secretion. **Results:** All the nanoparticles induced apoptosis, but only TiO_2_ NPs (alone or assembled into MSN) led to ROS and micronuclei. However, TiO_2_@MSN showed lower ROS and cytotoxicity with respect to the P25. Exposure to TiO_2_@MSN induced Th2-skewed and pro-fibrotic responses. **Conclusions:** Geno-cytotoxicity data indicate that TiO_2_@MSN are safer than P25 and MSN. Cytokine responses induced by TiO_2_@MSN are imputable to both the TiO_2_ NPs and MSN, and, therefore, considered of low immunotoxicological relevance. This analytical assessment might provide hints for NPs modification and deep purification to reduce the risk of health effects in the settings of their large-scale manufacturing and everyday usage by consumers.

## 1. Introduction

Until a few decades ago, microsized (approximately 0.1–10.0 µm) titanium dioxide (TiO_2_, titania) was incorporated in sunscreens as an inorganic ultraviolet (UV) filter [1]. In such form, its cosmetic profile was low due to its thick and chalky appearance on the skin. More recently, TiO_2_ has been prepared in the form of nanoparticles (<100 nm in size, TiO_2_ NPs), becoming one of the most produced nanomaterials, finding applications in a wide variety of technological fields, such as in cosmetics [2]. Indeed, TiO_2_ NPs are characterized by suitable bandgap energy leading to effective UV absorption exploited as a UV filter for sunscreen formulations. TiO_2_ NPs incorporated into sunscreen formulations are invisible to the naked eye after application on the skin, while remaining effective in photoprotecting against UV radiation [3]. Nowadays, according to European Union (EU) regulations, the maximum admitted total concentration of TiO_2_ (sum of micro and/or nanoform) in cosmetic formulations is 25 wt%. However, it is prohibited in applications that may result in exposure of the end user’s lungs by inhalation [4]. In addition to transparency on the skin, TiO_2_ NPs offer other advantages that make them cosmetically appealing, such as the absence of skin irritation and sensitization, non-comedogenicity, chemical stability, low reactivity in terms of toxicity profiles, and allergic reactions as well as a low cost [3,5]. However, TiO_2_ is a semiconductor material and, particularly in nanoparticle form, is a strong photocatalyst, mediated in the presence of light by formation of super-oxide anion radicals (O^−^_2_), hydroxyl radicals (•OH) and H_2_O_2_ [6,7] raising concerns about photomutagenesis [8]. From the point of view of applications, the photocatalytic activity of TiO_2_ NPs leads to mutually opposite effects: (i) beneficial effects when used in industrial applications, such as in organic waste and waste water treatment processes [9], self-cleaning surfaces [10] and as a potential tumor cell destructive agent [11]; and (ii) potential harmful effects when used in cosmetics as sun UV blocker, due to the photodegradation of the organic matrices present in sunscreen formulations [12] as well as the generation of reactive oxygen species (ROS) that can impact the cellular and genetic integrity of a living cell, possibly driving it toward apoptosis [13]. Oxidative stress is also responsible for genotoxic effects, even though DNA damage without generating ROS and the generation of ROS without DNA damage were reported [14]. The phototoxicity of the nanosized TiO_2_ filter and its inclusion in sunscreens has also raised safety questions, although toxicity concerns can only occur when TiO_2_ NPs are able to penetrate the *stratum corneum* entering the dermis [15]. The question of whether TiO_2_ NPs penetrate deeper levels of skin to any significant degree is not entirely resolved [16]: most of the currently available data suggest a lack of absorption across both intact and damaged (tape-stripped) skin [8] and a penetration only in the outermost corneocytes of healthy and psoriatic skin [17]. In order to minimize the formation of ROS, and to prevent potential cell damage when irradiated with UV light, different strategies were recently proposed. The use of non-semiconductor coating materials (such as silica), applied to the surface of a semiconductor, was demonstrated to be quite effective to limit its photocatalytic activity and to reduce the citotoxicity [13,18,19,20,21,22,23,24]. A different approach consists in the growth of the active UV filter into the pores of mesoporous silica nanoparticles (MSN). The control of the in situ growth of nanocrystalline particles into the pores of MSN has the advantage of a wide tunability in terms of pores and MSN sizes. The flexibility of MSN was exploited, for example, to reduce the citotoxicity and simultaneously improve the stability of metal halide perovskites [25,26] or to stabilize metastable crystalline phases [27]. In this context, TiO_2_ NPs embedded MSN (TiO_2_@MSN) were synthesized as a case study [20]. TiO_2_@MSN nanocomposite may have different properties with respect to its single NPs constituents [19], leading to unpredictable outcomes when it interacts with biological tissues. Therefore, immunotoxicity studies on TiO_2_@MSN, bare MSN and a commercial nano-TiO_2_ are crucial to assess its potential as a commercial UV filter and highly desirable to ensure the maximum protection of the skin in the perspective of sustainable nanotechnology.

In vitro immunotoxicity was observed in RAW 264.7 murine leukemic monocyte macrophages exposed to TiO_2_ NPs by simultaneous induction of immunocyte apoptosis and multiple toll-like receptors (TLRs) signaling through oxidative stress-dependent SAPK/JNK (Stress-activated protein kinases (SAPK)/Jun amino-terminal kinases (JNK)) and p38 mitogen-associated protein kinase activation [28]. Animal studies showed that TiO_2_ NPs can translocate to the lymph nodes by lymphatic vessels and can activate dendritic cells [29].

In vivo studies in rodents showed that topical, inhalant or intratracheal applied TiO_2_ NPs alone worsened atopic dermatitis, induced lung injury (Interleukin-4 (IL-4) independent) and T helper-dependent inflammatory responses through ROS production and apoptosis. Moreover, chronically inhaled TiO_2_ NPs induced pulmonary inflammation and fibrosis, increased expression NF-κB along with a large number of inflammatory and fibrotic cytokines in the lung. MSN resulted in being both non-toxic and non-inflammagenic, inducing only very low immune responses in splenocytes as determined by surface expression of activation markers and release of pro-inflammatory cytokines such as Interleukin-6, -12 and -1β [30]. In addition, MSN were not noxious to chronically administered animals [31]. Nevertheless, MSN are captured by phagocytic monocyte cells and potentially cause toxicity to the immune system by ROS overproduction and proinflammatory response. Some studies showed primary or secondary dose- and size-related genotoxic effects (DNA alkylation, apoptosis, DNA strands break and chromosomal aberrations, micronuclei) of silica nanoparticles in phagocytic cells and in animals [32]. An in vitro study on human lung epithelial cells A549 exposed to TiO_2_@MSN nanocomposites showed that the different ratio among silica and titania played a crucial role in the induced cytotoxicity [19]. In addition, TiO_2_/mesoporous silica nanotubes resulted in being biocompatible with mouse fibroblast cells, although still highly photocatalytic [33]. The aim of the present study was to evaluate aspects of genotoxicity and immunotoxicity of TiO_2_@MSN, compared to bare MSN and commercial nano-TiO_2_ NPs. To this end, investigations on these three systems of NPs concerned (i) the in vitro effects on cell metabolism/cytotoxic profile using the prototypical fibroblast-like murine L929 cells; and (ii) the ex vivo cytogenotoxic and immunotoxic responses using primary human peripheral blood mononuclear cells (PBMC) as target cells. The results suggest the use of TiO_2_@MSN as a potential UV nanofilter for cosmetic application.

## 2. Materials and Methods

### 2.1. Nanoparticle Synthesis and Characterization

MSN (≈100–150 nm, ordered pores, hexagonal symmetry, pore size ≈5 nm) were synthetized as described by Ma et al. [34]. Based on preliminary results [20], a TiO_2_@MSN nanocomposite (10 wt% of TiO_2_) was prepared by the impregnation method and fully characterized from a physicochemical point of view. For comparison, a commercially available nano-TiO_2_ Evonik Aeroxide^®^ P25 (Evonik, Essen, Germany) labelled as P25 was tested.

Verification of the SiO_2_/TiO_2_ ratio of the synthesized material was performed by instrumental neutron activation analysis (INAA) at the Triga Mark II reactor of Pavia University [35]. The SiO_2_/TiO_2_ ratio experimentally determined (10.8) was in good agreement with the theoretical one.

The absence of bacterial and mycoplasma contamination was checked by seeding 100 μL of 2000 μg nanoparticles/mL stock suspension of the nanoparticles onto non-selective agar plate and growth for 4 days at 37 °C, followed by polymerase chain reaction (PCR) testing with mycoplasma-specific primers/agarose gel electrophoresis/fluorescent dye UV-visualization. Potential interference of MSN and TiO_2_@MSN on absorbance of ultraviolet–visible (UV–vis) light and the ability to induce the formation of cell-independent product and scattering in these assays was checked by analyzing control samples containing particles without cells. NPs’ cytotoxicity was evaluated by two complementary colorimetric assays measuring either mitochondrial enzyme activity (MTS) and lactate dehydrogenase release in the culture medium (LDH), using conventional fibroblast-like murine L929 cells [21]. The first is based on the reduction of tetrazolium salt (MTS, yellow) to formazan (purple) by mitochondrial succinate dehydrogenase enzyme; the second is based on cytoplasmic lactate dehydrogenase activity and detection of the product in culture medium (Table 1).

### 2.2. Human Primary Mono/Lymphocytes

Human peripheral blood mononuclear cells (PBMC) were isolated by density gradient centrifugation on Lymphoprep medium (Axis Shield) [36] of freshly withdrawn ACD blood from a healthy donor. They were seeded in a 96-well plate (200,000 cells/well) in 200 µL complete culture medium (RPMI, 10% FCS), supplemented or not with the polyclonal activator phytohaemoagglutinin (PHA, 5 mg/mL) in presence of 0, 1, 25, 50, 100 µg NPs/mL in culture medium. Such NPs concentrations were obtained by dilution of a 10 mg NPs/mL stock suspension pre-filtered trough 22 µm nylon mesh filter (Millipore, Darmstadt, Germany). PHA is a polyclonal activator promoting agglutination by close contacts between cell membranes, hence stimulating cell division and metabolism, used as positive control for stimulation of (otherwise resting) human PBMC and induction of functional T-lymphocytes in vitro.

**Figure 1:**
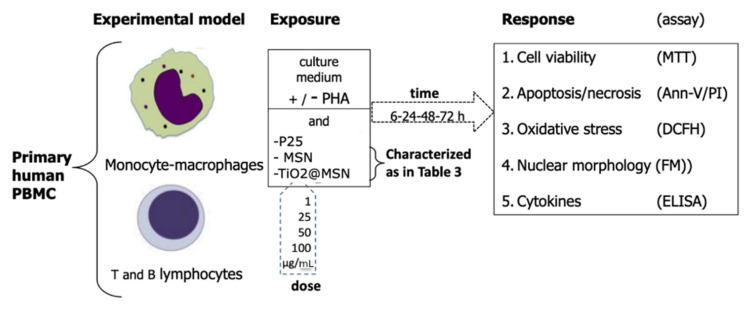
Study outline.

Under these conditions, lympho/monocytes (either unstimulated or PHA-activated), were used as target cells in vitro to evaluate the cyto/immunotoxicity after being induced by exposure for 6, 24, 48, 72 h in humified 5% CO_2_, 37 °C by TiO_2_@MSN, MSN and nano-TiO_2_ (Figure 1).

### 2.3. Cell Viability/Cytotoxicity

Cell viability was evaluated by the MTT colorimetric technique [37]. Briefly, 20 μL of the yellow tetrazolium (MTT (3-(4,5-dimethylthiazol-2)-2,5 diphenyl tetrazolium bromide) (Sigma-Aldrich, Milano, Italy, 5 mg/mL in phosphate-buffered saline (PBS)), was added to each well. The plates were incubated for 3 h at 37 °C 5% CO_2_, for reduction of MTT by metabolically active cells. The medium was then carefully removed and solubilization of formazan crystals (insoluble purple product of MTT reduction) 200 μL DMSO (Dimethyl Sulfoxide) was added to each well. The plates were placed on a shaker for 15 min in order to achieve the complete solubilization of the crystals and then the optical density of each culture supernatant was determined. The quantity of formazan product was measured by the amount of 540 nm absorbance, which is directly proportional to the number of living cells in culture. Changes in cell viability measured by MTT were compared to that of PBMCs in complete RPMI culture medium used for preparing NPs suspensions and cells exposure.

### 2.4. Apoptosis

Apoptotic cells were detected and discriminated from viable cells by fluorochrome conjugated-Annexin V that can bind to phosphatildylserine residues exposed on the outer layer only on damaged cell membranes [38]. Necrotic cells were identified as cells positively stained with the DNA-specific fluorescent dye propidium iodide (PI, Sigma-Aldrich, Milano, Italy). Lymphocytes (0.5 × 10^6^ cells) were washed with 1 mL of PBS, resuspended in 70 µL binding buffer (10 mM Hepes, 140 mM NaCl, 2.5 mM CaCl_2_, 0.1% BSA, pH 7.4), stained with 5 µL FITC-conjugated Annexin V (Valter Occhiena, Torino, Italy) and 0.5 µg/mL propidium iodide for 15 min, at room temperature, in the dark. As positive control, 1 mM H_2_O_2_ was added to unexposed cells and incubated for 3 h. Samples were analyzed immediately after staining by flow cytometry using the FASCanto cytofluorimeter (Becton Dickinson, Milano, Italy) and the acquired data (10,000 events within the viable cell gate, per sample) were analyzed with FacsDiva software v 6.1.3 (BD). Annexin V-positive/PI negative cells are considered apoptotic, whereas PI positive are considered as necrotic.

### 2.5. Oxidative Stress

Oxidative stress was evaluated by the level of intracellular ROS measured using the dye dichlorodihydrofluorescin diacetate (DCFH-DA) [39]. Briefly, 2 × 10^5^ PBMC per well were seeded in a 96-well cell culture plate and treated with or without nanoparticles. After 24 h, cells were extensively washed with PBS and incubated with 10 µM DCFH-DA in PBS, for 30 min, at 37 °C. As positive control, 1 mM H_2_O_2_ was added to another duplicate of cultures. Fluorescence of the oxidized form of DCFH-DA (DCF) was measured using a FacsCanto cytofluorimeter (excitation wavelength: 485 nm; emission wavelength: 530 nm). For each sample, 10,000 events within the viable cells gate were acquired. Each condition was tested in quadruplicate.

### 2.6. Nuclear Staining

The nuclear morphology was examined using a confocal fluorescence microscope (IX71, Olympus, Düsseldorf, Germany) by staining cells with a fluorescent DNA-binding dye. After incubation in media containing different concentrations of NPs, the cultured PBMCs were treated with 100 mg/mL of the fluorescent DNA-biding dye 488 Green (Sigma-Aldrich, Merkgroup, Milano, Italy) and then observed.

### 2.7. Cytokines Secretion

PBMC (500,000 cells per well in 0.5 mL culture medium + PHA) were incubated for 48 h with nanoparticles (25 µg/mL, non cytotoxic concentration) in triplicate wells and, in parallel samples, no NPs were added to set the baseline levels. Cell culture supernatants were harvested and stored at −70 °C until analyzed for the quantifications of different cytokines (Interleukin-17 (IL-17), IL-23, Interferon- γ (IFN-γ), IL-10, IL-13, IL-1-β, IL-2, IL-6, Tumor Necrosis Factor- α (TNF-α) by enzyme-linked immunosorbent assay (ELISA) MAP Human Cytokine reagents and the Milliplex platform (Millipore, Darmstadt, Germany), according to the protocol provided by the manufacturer.

### 2.8. Statistical Analysis

All experiments were done in triplicate and the results were presented as mean ± standard error of the mean (SEM). To evaluate the differences between conditions of culture relatively to time and dose of exposure and treatment data were analyzed by two-way analysis of variance (ANOVA) followed by Bonferroni post-test using GraphPad Prism software version 4.00 (GraphPad Software, San Diego, CA, USA). Differences were considered significant at *p* < 0.05.

## 3. Results

### 3.1. In Vitro Exposure of Peripheral Blood Mononuclear Cells (PBMC)

#### 3.1.1. Cell Viability

**Figure 2:**
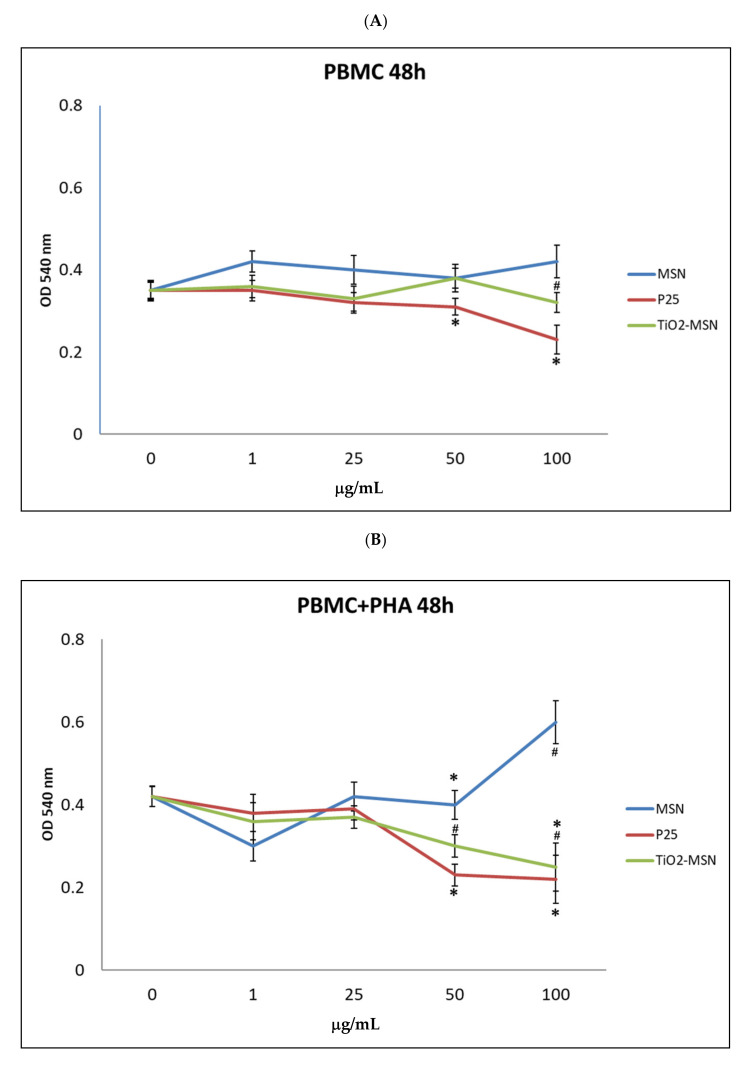
MTT (3-(4,5-dimethylthiazol-2)-2,5 diphenyl tetrazolium bromide) viability assay. (**A**) without phytohaemoagglutinin (PHA) and (**B**) with PHA. Significance values: * or # = *p* < 0.05; error bars represent the standard error of the mean.

No cytotoxicity was observed after 6 and 24 h of incubation of TiO_2_@MSN, MSN and P25 for both PHA-stimulated and unstimulated PBMC (results not shown). After 48 h, different cytotoxicity profiles were found in PHA-activated or unstimulated PBMC. In PHA-stimulated PBMC, exposure to increasing concentrations of MSN were not associated with proportional changes of cell viability (−25% at 1 µg/mL, +33% at 100 µg/mL). Decrease of viable cells (−40%) was associated with the exposure to the highest doses (50 µg/mL and 100 µg/mL) of P25 and TiO_2_@MSN (Figure 2).

#### 3.1.2. Apoptosis and Necrosis

**Figure 3:**
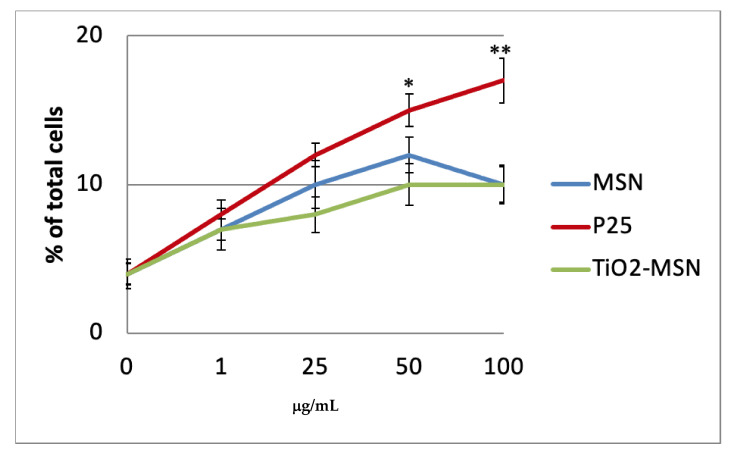
Apoptosis detection of resting peripheral blood mononuclear cells (PBMC) by annexin V staining after 6 h culture without PHA. Significance values: * *p* < 0.05, ** *p* < 0.005; error bars represent the standard error of the mean.

PHA-unstimulated PBMCs ex vivo in culture incubated with TiO_2_@MSN, MSN and nano-TiO_2_ for up to 48 h maintained a high rate of viable cells (90–95%); the apoptosis rate for P25 is significantly higher at 50 and 100 µg/mL (approx. 14% and 17%, respectively) compared to TiO_2_@MSN (approx. 10%) (Figure 3). After 72 h, as expected for primary cells ex vivo, the percent of viable cells decreased to 80–85% (data not shown).

Viability of PHA-activated PBMCs culture showed a more complex outline. The three types of NPs induced dose-dependent apoptosis within 6 h (independently from exposure to PHA).

**Figure 4:**
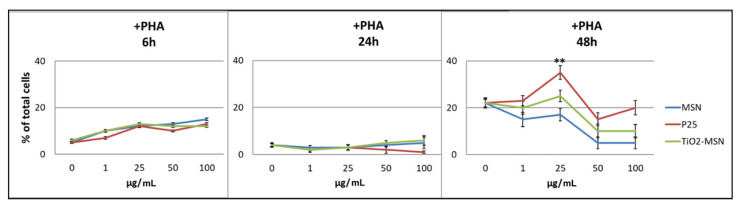
Apoptosis detection of PHA-stimulated PBMC by Annexin V staining. Significance values: ** *p* < 0.005; error bars represent the standard error of the mean.

After 24 h, a lower degree of apoptotic cells (3%) was detected among PHA-activated PBMCs. After 48 h, all three curves were almost congruent, but not dose-dependent, with the highest apoptosis shown by only P25 at 25 µg/mL (Figure 4).

**Figure 5:**
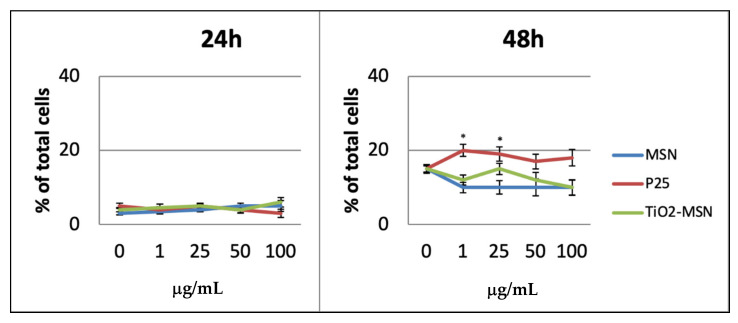
Necrosis detection by propidium iodide staining. Significance values: * *p* < 0.05; error bars represent the standard error of the mean.

Necrosis was not observed after 6 h and appeared in low percentage after 24 h in any condition of exposure. After 48 h the highest rate of necrotic cells (approx. 18%) was associated with P25, while approx. 10% were detected for TiO2@MSN and MSN (Figure 5).

#### 3.1.3. Oxidative Stress

**Figure 6:**
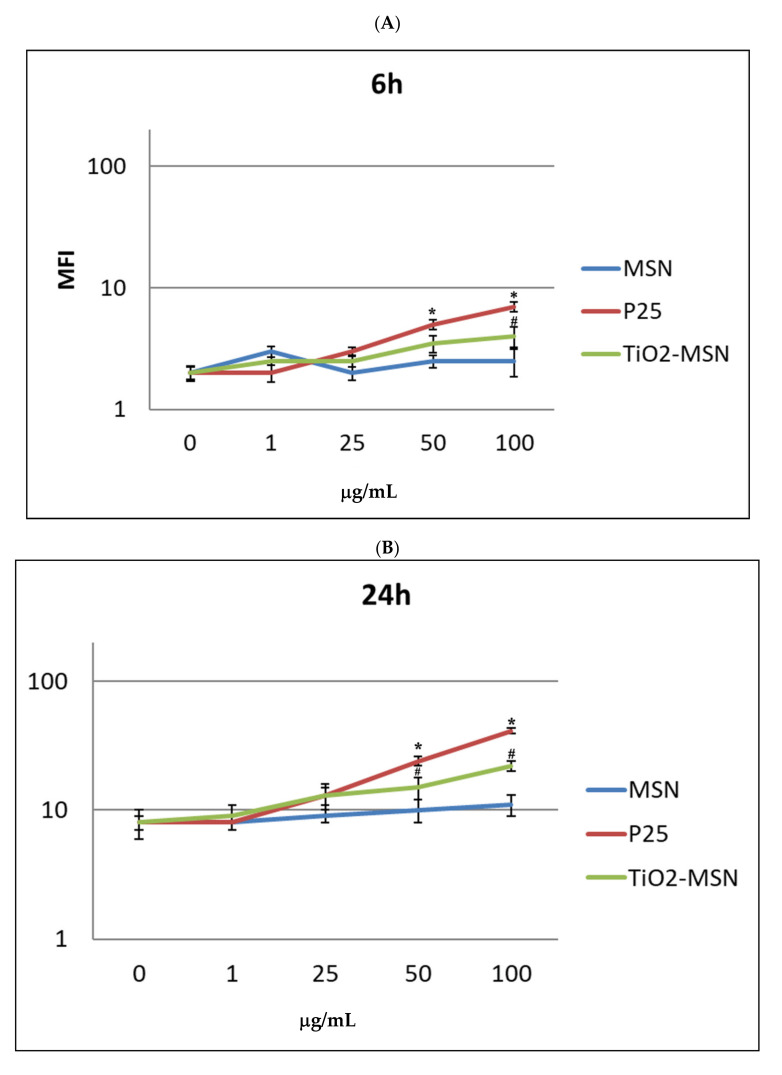

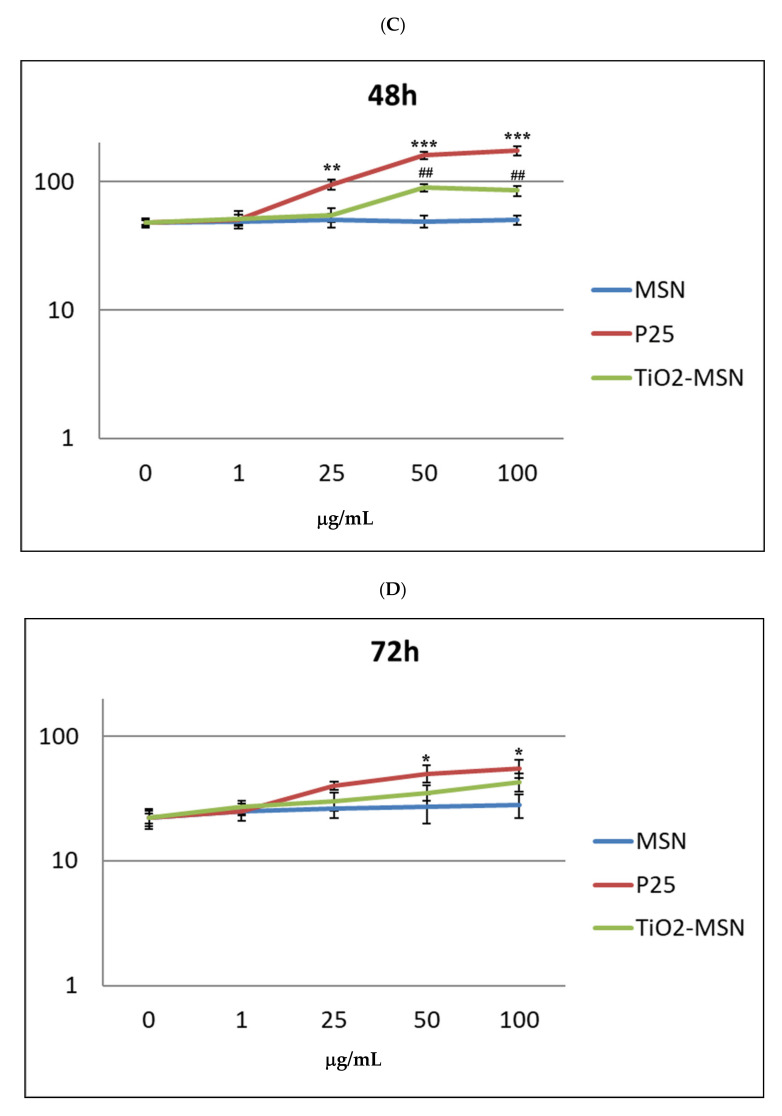
Reactive oxygen species (ROS) detection by DCFDH probe at increasing time of exposure: (**A**) 6 h. (**B**) 24 h. (**C**) 48 h. (**D**) 72 h. Significance values: * or #: *p* < 0.05; ** or ##: *p* < 0.005; ***: *p* < 0.0005; error bars represent the standard error of the mean.

ROS increased in in vitro PBMC exposed to P25 and TiO_2_@MSN in a time- and dose-dependent manner from 6 h (Figure 6) to 48 h.

Remarkably, 1 to 50 μg/mL of P25 determined the sharpest increase of ROS, compared with equal doses of the other compound. MSN exposure was associated with increase of ROS level by time, but conversely, was independent of dose. In any condition, a proportional reduction of ROS levels was measured after 72 h. However, at this timepoint extensive cell damage appeared by optical microscope observation (40×) (not shown). As expected, PHA-stimulated PBMC showed a generally higher ROS level (not shown), compared to unstimulated PBMC. Notably, P25, in any time and culturing condition induced the highest level of ROS. The two highest doses of TiO_2_@MSN were associated with induction of a lower level of ROS at 24 h and 48 h.

#### 3.1.4. Nuclear Morphology

**Figure 7:**
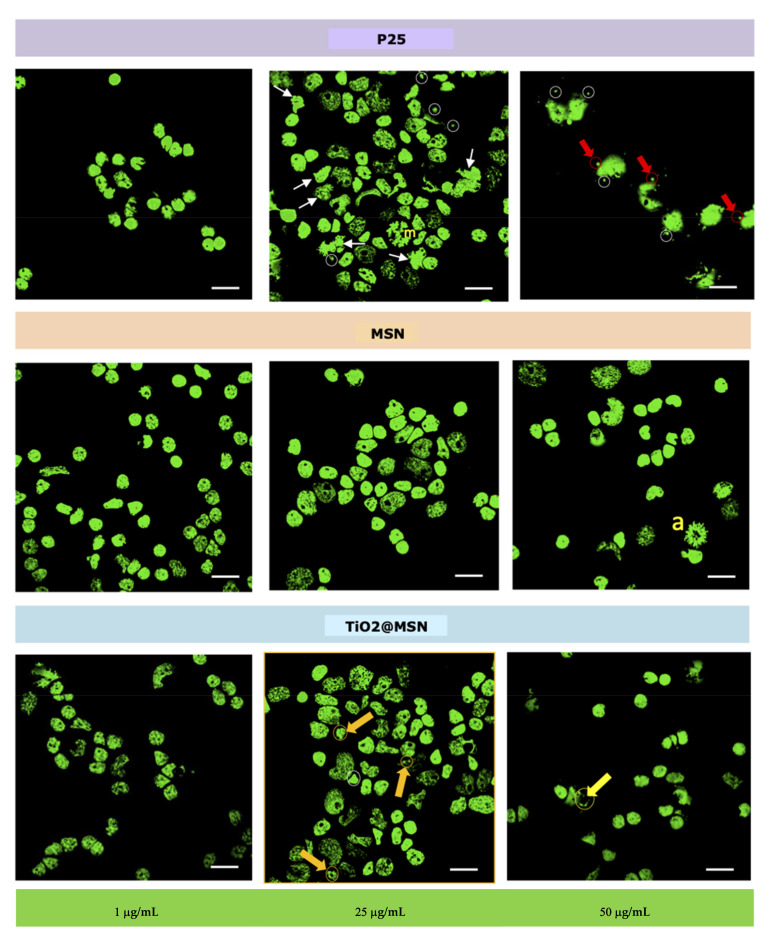
Nuclear morphology. Bright micronuclei (MN, circles), nuclear anomalies (white arrows). Nuclear buds (NBUDs, red arrows) and chromatin bridges (yellow arrow). Dividing nuclei were observed (m = metaphase; a = anaphase).

Bright nuclei (interphase condensed chromatin) with organized nucleoli as well as enlarged nuclei (replicating chromatin), typical of viable cells, were visible in samples exposed to the lowest concentration (1 µg/mL) of either of the three types of NP. Beside those, weakly colored nuclei (i.e., less organized chromatin) characteristic of cells undergoing necrosis, were visible. Furthermore, bright micronuclei (MN, circles), i.e., small chromatin formations clearly distinguished from the nucleus, adjacent to it, with same coloration and size between 1/16 and 1/3 of the mean diameter of the nucleous [40], were apparent in the samples exposed to 25 µg/mL of P25 or TiO_2_@MSN, with a higher frequency for the former. Nuclear abnomalies (white arrows) were evident only in samples exposed to P25 (25 µg/mL). At the highest concentration (50 µg/mL), nuclear buds (NBUDs, red arrows) and chromatin bridges (yellow arrow) were also present. Dividing nuclei were observed in the sample exposed to 25 µg/mL P25 (m = metaphase) and 50 µg/mL MSN (a = anaphase) (Figure 7).

#### 3.1.5. Cytokines Profile

Unstimulated PBMC (no PHA) produced barely detectable (IL-2, IL-6, IL-1β, TNF-α, IL-4, IL-10, IL-23) or undetectable (IL-17 and IFN-γ) cytokine proteins in the culture medium upon exposure to TiO_2_@MSN, MSN or P25. The addition of T-cell polyclonal activator PHA to PBMC induced a strong increase in the secretion of IL-2, IL-4, IL-10, IL-17, IL-23, and IFN-γ and no quantitative changes were observed in IL-6, IL-1β and TNF-α expression, compared to the unstimulated control.

**Figure 8:**
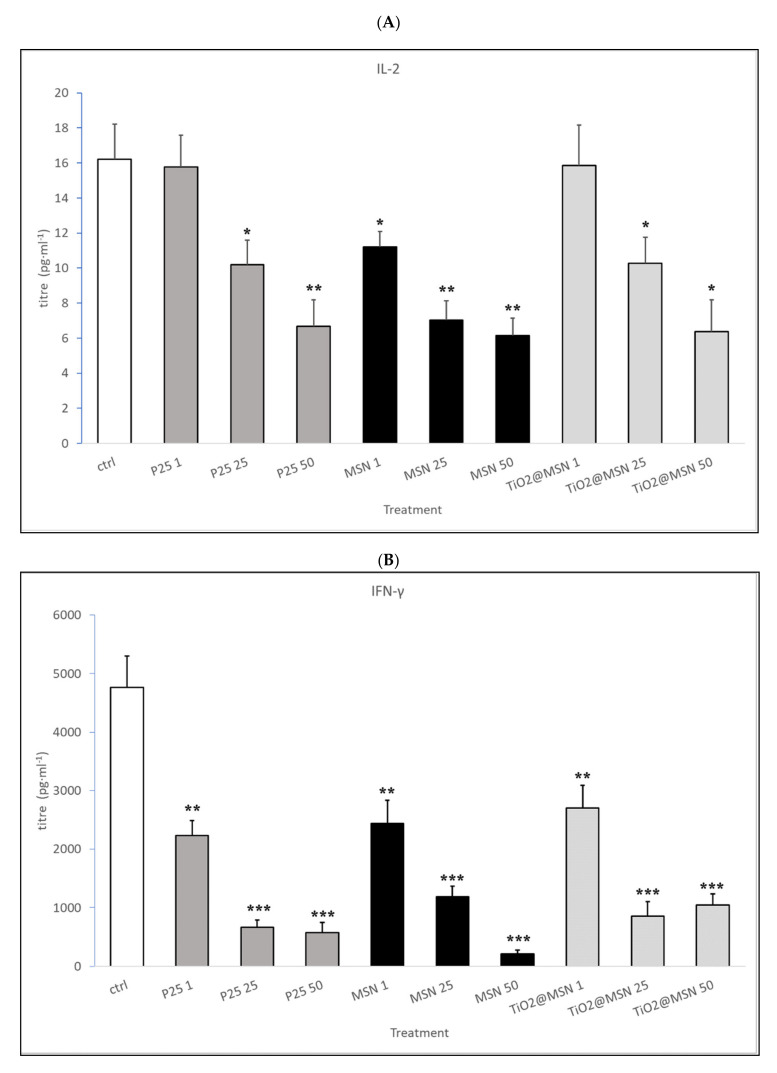

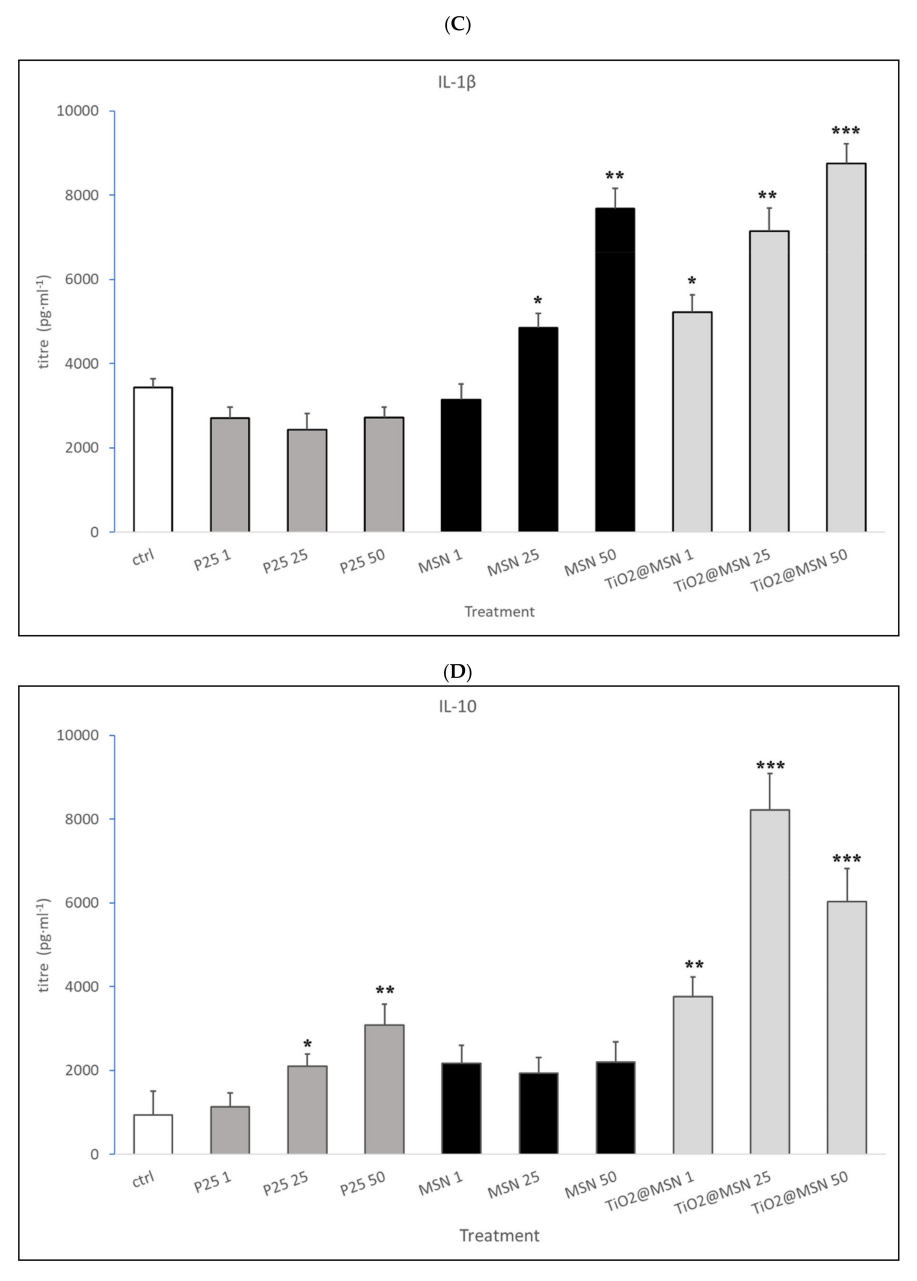

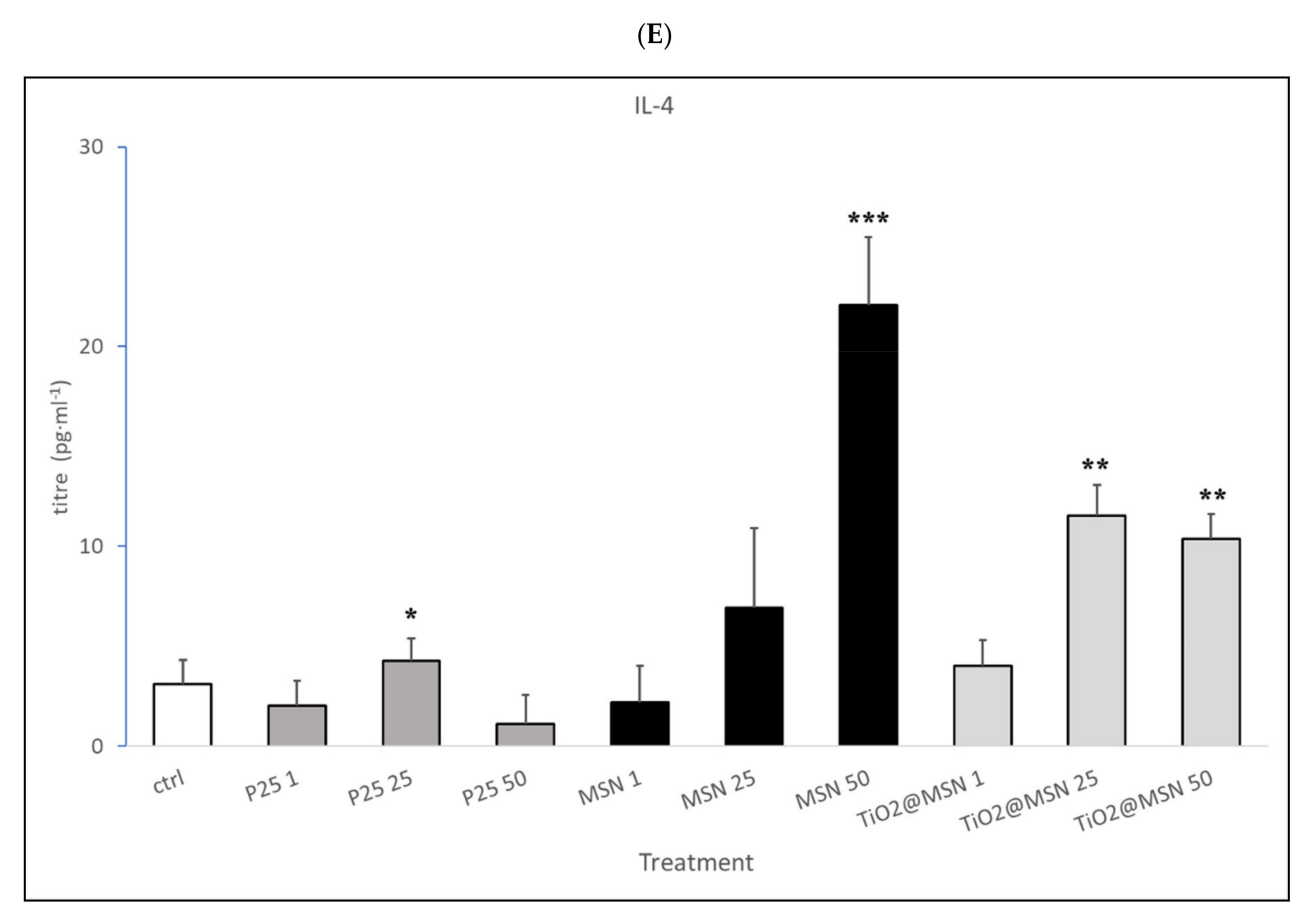
Secreted cytokines pattern: titer of (**A**) IL-2; (**B**) INF-γ; (**C**) IL-1β; (**D**) IL-10; (**E**) IL-4. Significance values: * *p* < 0.05, ** *p* < 0.005, *** *p* < 0.0005; error bars represent the standard error of the mean.

Addition of the three types of NP in the culture medium produced the following effects on the level of cytokines secreted by PHA-stimulated PBMCs (Table 2 and Figure 8):

(i) MSN exposure was associated with strong increase of IL-1β and IL-4, a decrease of IL-2 and IFN-γ levels, in a dose-dependent manner. IL-17 and IL-23 were down-modulated while no significant changes in IL-6, TNF-α, IL-10 were detected (data not shown).
(ii) P25 determined a decrease of IL-2 and IFN-γ, increase of IL-4 (at non cytotoxic concentrations, 1–50 µg/mL) and an obvious dose-dependent increase of IL-10, and TNF-α. IL-17 and IL-23 were downmodulated, whereas IL-6 and IL-1β were not affected (data not shown).
(iii) TiO_2_@MSN induced dose-dependent reduction of IL-2 and IFN-γ, particularly high spikes of TNF-α and dose-dependent increase of IL-1β and IL-10. Moreover, it induced a dose- and time-dependent fluctuating levels of TNF-α and IL-4, an increase of IL-17 and IL-23 as well as no significant change of (limited) IL-6 (Figure 8).

## 4. Discussion

TiO_2_ NPs are an essential nanomaterial for numerous technological applications. In particular, they are appealing as protective UV filter in solar sunscreens, use of which would entail frequent and pervasive cutaneous (skin and hair) treatment with TiO_2_ NPs for over-exposed or inherently under-protected people, such as infants, the elderly and outdoor workers.

Regrettably, toxicological data gathered so far address potential pitfalls of these NPs related to its chemistry and size. In human skin cell-based experiments, often characterized by high doses of exposure, TiO_2_ causes oxidative stress and DNA damage [41] and, extracted from sunscreens, induces apoptosis of exposed UVA-irradiated cells, an effect that is dampened by pre-coating [42]. Furthermore, TiO_2_ NPs are phagocytized by monocytes/macrophages that consequently display oxidative stress and genotoxic effects [43]. In mouse brain microglia, at non-cytotoxic concentrations, TiO_2_ NPs form cytoplasmic aggregates and stimulate ROS [44]. In chronically exposed rodents, TiO_2_ induces pro-inflammatory cytokines and fibrosis of the lung [45], and TiO_2_ NPs appear to play a role in the onset and aggravation of allergies through a cyto-genotoxic mechanism [46]. Also, human lymphocytes, either from healthy individuals or with respiratory disease, are damaged by TiO_2_ NPs at the DNA level [47]. Human studies, regarding professionally exposed worker to TiO_2_ (nanoparticles), show oxidative damage of nucleic acids of Ti-containing exhaled breath condensate samples [48] and increased oxidative stress and overexpression of inflammatory and fibrogenic cytokines [49]. However, the aforementioned studies are limited in numbers, jagged/fragmentary and not specifically addressed to highlight TiO_2_ NPs in humans.

Hence, to improve the efficiency of TiO_2_ NPs as a UV filter and, concurrently, its biosafety, we synthetized a nanotitania composite by growing TiO_2_ NPs inside MSN. In fact, MSN are largely used in therapeutic and diagnostic applications for their high surface area, large pore size, good biocompatibility and biodegradability, and stability in aqueous dispersions [50]. TiO_2_@MSN was submitted to key geno-immunotoxic assessment by comparative testing against MSN and P25 in human PBMC, using a combination of damage/defense responses of immune cells against noxious agents (i.e., apoptosis, ROS, nuclear morphology evaluation and released cytokines detection).

In previous studies, we showed that primary human PBMC were useful for ex vivo evaluation of the genotoxic and immunotoxic potential of NPs [51,52,53,54,55]. Also, PBMC testing was proposed for monitoring the health protection of potentially exposed workers at higher risk of asthma and atopic dermatitis [56] and as a diagnostic biomarker [57]. Recently, the pre-clinical evaluation of cytokines released by PBMC in vitro has been recommended by the FDA (Food and Drug Administration) for large (protein) therapeutic molecules [58]. In addition, we have shown that PBMC produce ROS, undergo apoptosis and characteristic dysregulation of cell cycle and cytokine expression when exposed to NPs [52,53,54,59,60,61,62,63,64,65,66].

Apoptosis occurs as a defence mechanism at a low dose preventing genotoxic mutation from becoming mutagenesis [67]. Moreover, nuclear abnormalities was detected as signs of genetic damage caused by NPs [68]. Amongst these are micronuclei, acentric fragments or entire chromosomes unable to migrate to the poles during cell division, and nuclear buds, that instead remain linked to the nucleus through a “bridge” of nucleoplasmic material [40].

Abnormal cytokine patterns can reflect genetic dysregulation and cancer [69] even ending in death (cytokine storm, cytokine release syndrome [70]). The expression of cytokines is firstly regulated at the transcription level through transcription factors that respond to signalling pathways activated by pathogen-derived ligands or endogenous inflammatory mediators [69]. Notably, NPs can mimic pathogens and bind surface receptors able to trigger those pathways [60]. Conversely, mutation (or inhibition) of a single transcription factor (TF) binding site can upheaval cytokine expression and lead to immune disorders [69]. All the aforementioned were detected in our study for either of two TiO_2_-based nanoparticles tested, and for MSN. Although the exact mechanism(s) of TiO_2_ NPs genotoxicity remain to be determined, oxidative stress might initiate it as suggested by the parallel increase of ROS. Nano-TiO_2_-induced genotoxicity could rely also on the inhibition of DNA base excision repair systems [71], known to operate when metal(oxide)-NP-based DNA damage occur [72].

It cannot be excluded that soluble compounds from aggregates of TiO_2_ NPs might have contributed to the observed effect, but these appear to be sensibly reduced by the growth of TiO_2_ NPs inside MSN.

ROS were detected also in cells exposed to the two highest concentrations of TiO_2_@MSN and, since MSN alone do not induce as much ROS as the TiO2@MSN, the geno-cytotoxicity appears to be mediated by nano-TiO_2_.

Our findings strongly suggest that “typical” ROS-induced genotoxicity of TiO_2_ NPs is abrogated when it is incorporated inside MSN nanopores and that, in such a form, it is associated with higher concentrations corresponding to aggregates formation; also, the biological modifications associated with TiO_2_@MSN exposure are detectable when the activation of the target lymphocytes is turned on. If confirmed, the use of MSN as a scaffold might represent a way to lower the toxic potential of TiO_2_ NPs and others.

The higher toxicity observed for TiO_2_@MSN might be related to the formation of larger aggregates observed when dispersed in culture medium supplemented with serum, a phenomenon being underlined as the most relevant in the assessment of nanoparticles toxicity and potential human effects. On the other hand, residual toxicity of the novel nanomaterial could be due to titania impurities, and chemically instability and loss of morphology in culture medium.

All three samples analyzed downmodulate IL-2 and IFN-γ cytokines. Likely, this reflects suppression of T helper 1 (Th1) cells that are central players in specific acquired immune response against microbial pathogens and tumor cells. On the other hand, over-secreted IL-4 might critically stimulate Th2 cells, physiologically involved in the fight against parasites but, together with B-cells, also targets of this cytokine, leading to detrimental allergic and fibrogenic responses too. Beside those shared effects, MSN and TiO_2_ NPs appear to display polarized behaviors, although hindering each other. In fact, MSN-containing NPs are associated with the increased release of IL-1β, as elsewhere described [63], known to be produced by and activate monocytes/macrophages to capture and annihilate intruding particulate matters generating inflammation in situ. Instead, P25 and TiO_2_@MSN appear to stimulate IL-10 and, therefore, might favor immune tolerance, antigen presentation and fibrosis mediated by responding regulatory T cells, antigen processing cells and epithelial cells, respectively.

## 5. Conclusions

This study shows that the novel nanocomposite TiO_2_@MSN, with improved physicochemical properties of TiO_2_ NPs, also shows improved biocompatibility in terms of lower cyto-genotoxic effects. Moreover, the observed Th2-skewed and pro-fibrotic responses upon acute exposure to TiO_2_@MSN are both likely imputable to the non-encapsulated precursor of TiO_2_ NPs, when analyzed comparatively. Furthermore, the observed cytokine pattern induced by TiO_2_@MSN is not dissimilar and typical of that of any other particulate material [63] and, therefore, to be considered of low immunotoxicological relevance. Collectively, this study shows that TiO_2_@MSN represents an advanced TiO_2_-based nanocomposite material suitable for bio-applications.

The pre-production evaluations described here based on complex interplaying human cellular populations along with the assessment of the true “biologic” NPs size allows rapid monitoring of innovative NPs biocompatibility. This analytical assessment also might provide hints for NPs modification (i.e., by encapsulation, anti-oxidant molecules etc.), and deep purification to reduce the risk of health effects in the settings of their large-scale manufacturing and everyday usage by consumers.

## Figures and Tables

**Table 1:** Physicochemical characterization and evaluation of contaminants and cytotoxic activity of TiO_2_@MSN.

Characterization	Analytical Method	Result
SiO_2_/TiO_2_ ratio	INAA (Instrumental Neutron Activation Analysis)	10.8
Physicochemical	BET/BJH (Brunauer, Emmett, Teller (BET)/Barrett, Joyner, Halenda (BJH) Determination of Specific Surface Area and Pore Size Distribution)	<S.A._BET_; <Dp; <Vp
	FT-IR (Fourier Transform Infrared Spectroscopy)	No variations of note
	XRD (x-ray diffraction)	Diffraction peaks: size nanopores ≈ 5 nmAnatase phase
	TEM (Transmission electron microscopy)	Size TiO_2_ NPs in MSN ≈ 5 nm
	UV-vis DR (Diffuse Reflectance)	Absorption band: 200–360 nm
	DLS (Dynamic Light Scattering)	Instability
	PCR (Polymerase Chain Reaction)	No mycoplasma contamination
Manufactured NP sample suitability (for further cytotoxicity assays)	MTS/LDH (Lactate Dehydrogenase)	No absorption No interference with detection reaction by TiO_2_@MSN Scattering activity: yes
	MTS ((3-(4,5-dimethylthiazol-2-yl)-5-(3carboxymethoxyphenyl) -2-(4-sulfophenyl)-2H-tetrazolium)	4 h → 48 h > MSN

**Table 2:** Dose-dependent cytokine patterns associated with exposure of PBMC to TiO_2_@MSN, MSN and P25 and their target cells of the immune system.

Samples	Cytokine Tested	Effect on Extracellular Eytokine Level	Types of Immune Cells Involved	Role
TiO_2_@MSN P25 MSN	IL-2	Decrease	Pan T cells	Acquired immunity
TiO_2_@MSN P25 MSN	IFN-γ	Decrease	T helper 1	Specific immune response against pathogens and Tumor cells
TiO_2_@MSN P25 MSN	IL-4	Increase	T helper 2, monocytes	Immune response against parasites Allergy Fibrosis
TiO_2_@MSN SN	IL-1β	Increase	Monocytes/macrophages, NK cells (and neutrophils)	Innate immunity against microorganism, tumor cells and particulate matters. Antigen presentation
TiO_2_@MSN P25	IL-10	Increase	Regulatory T cells, dendritic cells	Immunological tolerance Professional antigen presentation Anti-parasite response Fibrosis

## Data Availability

The data presented in this study are available on request from the corresponding author.
